# Hemodynamic Assessment of Dual Obstructive Left Ventricular Assist Device Lesions

**DOI:** 10.7759/cureus.17180

**Published:** 2021-08-14

**Authors:** Mahwash Kassi, Andrew N Rosenbaum, Abdallah El Sabbagh, Barry Boilson, Atta Behfar

**Affiliations:** 1 Department of Cardiology, Houston Methodist, Houston, USA; 2 Department of Cardiovascular Medicine, Mayo Clinic, Rochester, USA

**Keywords:** lvad, inflow, outflow, obstruction, dynamic inflow obstruction, intra-cardiac echocardiography, hemodynamic catheterization

## Abstract

Obstructive left ventricular assist device (LVAD) lesions are uncommon but are being increasingly recognized, particularly with the increased use of advanced imaging modalities. While heart failure symptoms and LVAD power fluctuations have a broad differential, obstructive lesions in the LVAD circuit should be considered. We present a unique case of a patient supported on HeartWare HVAD (Medtronic Inc., Dublin, Ireland) therapy, who experienced postural dizziness with objective orthostatic hypotension and occasional ventricular tachycardia. With fluctuations in LVAD flow and power, a CT scan with three-dimensional reconstruction was obtained showing outflow graft kinking. The patient was brought to the cardiac catheterization laboratory for investigation and consideration of outflow graft intervention. However, intracardiac echocardiography revealed the presence of an inflow cannula obstruction with position changes and catheter interrogation involving the outflow cannula suggestive of a gradient across the kinked area as an unlikely cause for the presentation. This case highlights the importance of a thorough interrogation for obstructive lesions in the setting of heart failure symptoms, particularly postural symptoms, in a patient on LVAD therapy, even when not identified on routine echocardiography.

## Introduction

Among hemodynamic complications related to left ventricular assist device (LVAD) therapy, obstructive lesions can be some of the most difficult to manage. Kinking in the outflow cannula is increasingly being recognized, courtesy of advanced imaging modalities, including three-dimensional (3D) cardiac CT reconstructions [1,2]. Treatment may involve stent placement or surgical revision, so accurate hemodynamic assessment of the outflow cannula is important to confirm findings by imaging [3-5]. There are no published guidelines or literature regarding the appropriate technique or parameters for this type of assessment. We present a complex case of outflow graft kinking and dynamic inflow obstruction.

## Case presentation

A 69-year-old gentleman with ischemic cardiomyopathy was implanted with a HeartWare HVAD (Medtronic Inc., Dublin, Ireland), as destination therapy. The outflow cannula graft was anastomosed to the descending aorta due to a porcelain ascending aorta. His postoperative period had been uneventful. Due to poor hemodynamics on pulmonary artery catheter monitoring, including mild biventricular filling pressure elevation and a cardiac index below 2.0 L/min/m2, his LVAD speed was set to 3000 revolutions per minute (RPM). However, in the recovery phase after implant, the patient had recurrent dizziness and ventricular tachycardia, preventing rehabilitation.

Given the debilitating symptoms, examination and echocardiography were undertaken to understand the pathophysiologic alterations. At 3000 RPM, with the patient lying down, blood pressure was 106/79 mmHg (mean 89 mmHg). Upon sitting, blood pressure dropped to 78/68. HVAD waveforms demonstrated a sawtooth pulsatility pattern in concert with a drop in power (5 W to 4.7 W) and estimated flow (3.7 L/min to 3.0 L/min). LVAD parameters and blood pressure normalized when he returned to a laying position. By echocardiography, the left ventricular end-diastolic dimension (LVEDD) decreased from 64 mm at baseline to 44 mm with sitting. The outflow cannula was poorly visualized.

While dehydration or low preload could have explained the apparent orthostatic findings, his examination was consistent with euvolemia. Furthermore, his right ventricular function was preserved on echocardiography. At this juncture, the pathogenesis of the intermittent symptoms was felt most likely due to inflow cannula obstruction.

**Figure 1:**
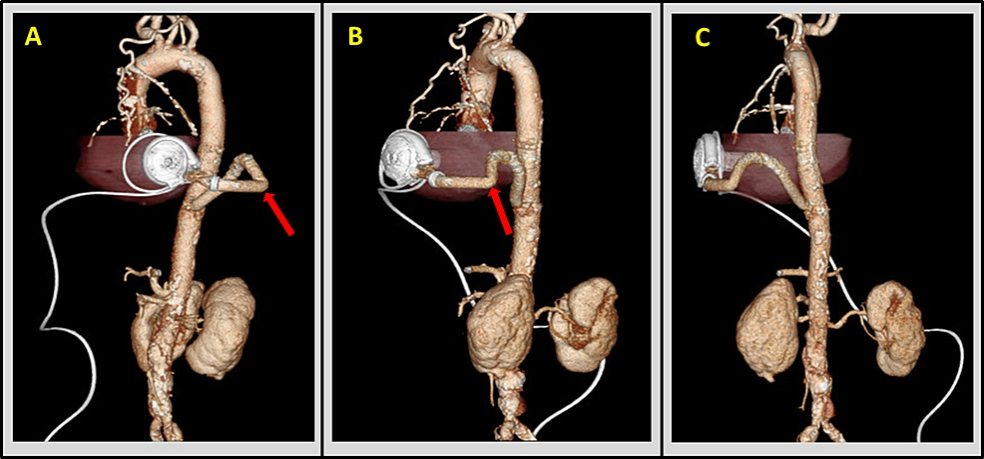
CT scan with 3D reconstruction of HVAD in situ ECG-gated CTA with 3D reconstruction of HVAD and outflow graft-to-descending thoracic aorta shown in oblique lateral (A), sagittal (B), and posterior (C) imaging planes, demonstrating acute angulation of approximately 90 degrees in the mid-graft (red arrows). ECG - electrocardiographic; CTA - computed tomography angiography; 3D - three-dimensional.

However, given the atypical anastomosis and postural symptoms, evaluation of the outflow graft was felt to be warranted. Therefore, a CT angiogram with 3D reconstruction was obtained, wherein a site of outflow graft kinking was observed (Figure 1). The inflow cannula orientation was appropriate. Findings raised the possibility that kinking of the outflow graft may be responsible not only for abnormal baseline hemodynamics (requiring a speed of 3000 RPM) but also that unkinking of outflow graft with positional change may be partially responsible for suction with positional change. Therefore, outflow graft intervention could facilitate improvement in hemodynamics at rest and allow for a reduction in LVAD speed, which would improve the risk of suction events. Given the uncertainty of hemodynamic effects of the outflow graft kinking and the possible need for outflow graft intervention, the patient was brought to the cardiac catheterization laboratory.

**Figure 2:**
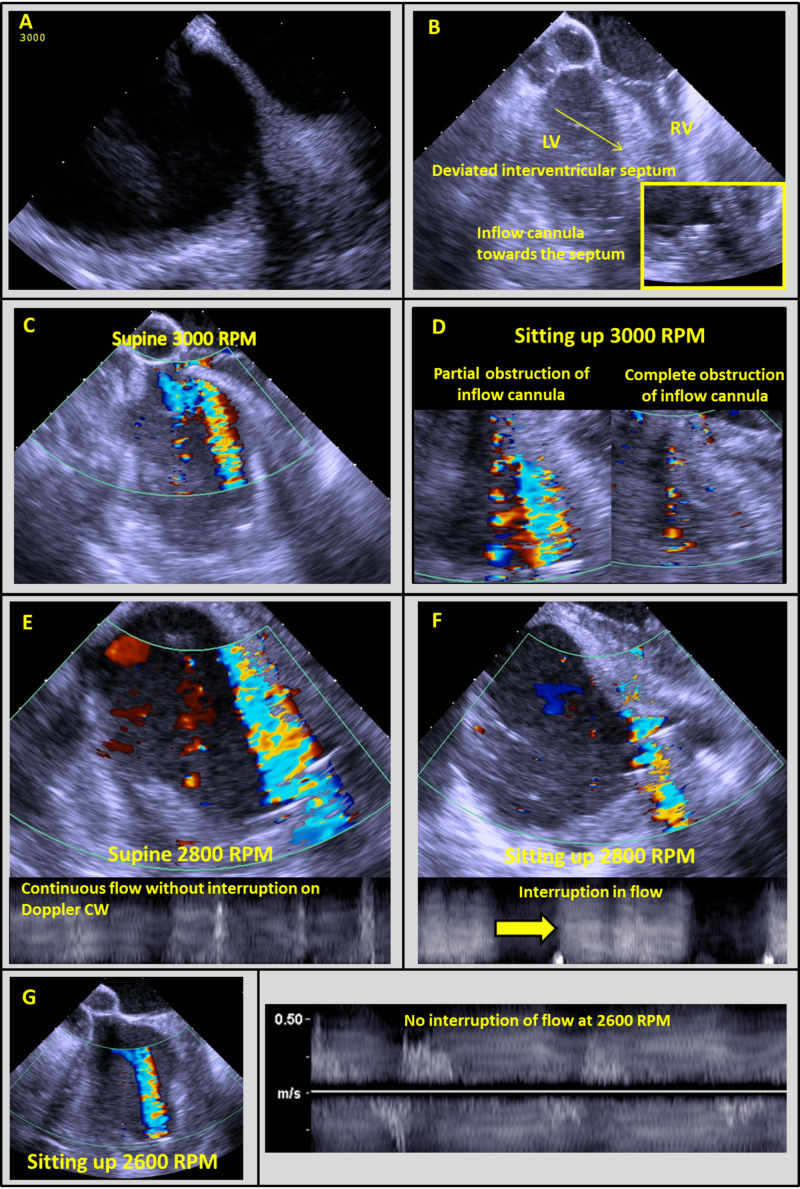
Intracardiac echocardiographic evaluation of HVAD inflow obstruction Initial evaluation demonstrating midline interatrial septum (A) and rightward interventricular septum as well as septal-oriented inflow cannula (B). Supine imaging at 3000 RPM showed normal flow (C), which was obstructed after sitting (D). Similar results were seen at 2800 RPM and inflow cannula CW demonstrated cessation of flow with sitting (E, F). At 2600 RPM in the setting position, no diminishment in the flow was seen by color flow or CW interrogation (G). RPM - revolutions per minute; CW - continuous wave.

Intracardiac echocardiography (ICE) was utilized given limited transthoracic images. At 3000 RPM, there was no aortic valve opening. The interatrial septum was midline and the interventricular septum was deviated to the right (Figures 2A, 2B). The inflow cannula was septal-facing but normal flow was observed (Figures 2B, 2C). However, in a sitting position, there was suction with interruption of flow in the inflow cannula (Figure 2D), which also occurred at 2800 RPM (Figures 2E, 2F). At 2600 RPM, no suction occurred with sitting (Figure 2G).

**Figure 3:**
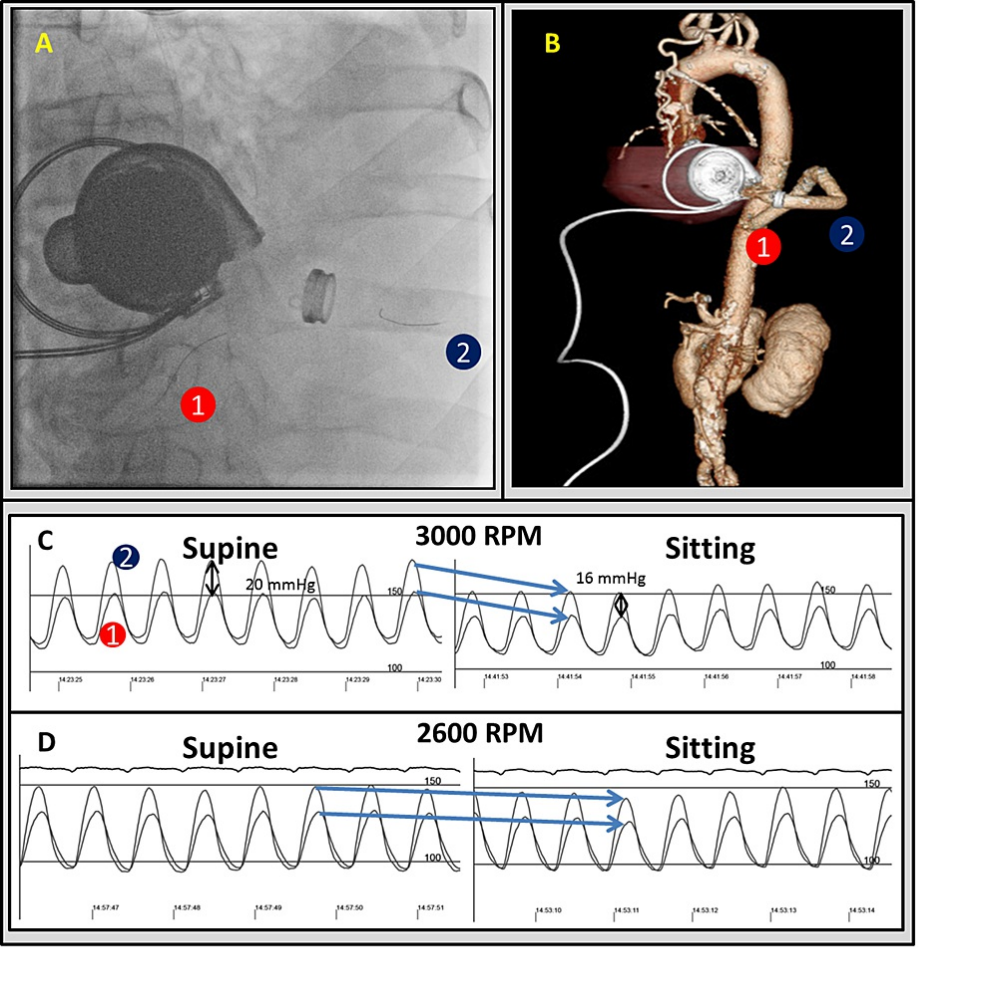
Hemodynamic evaluation of outflow and dynamic inflow obstruction Fluoroscopic image showing the location of the aortic catheter and high-fidelity micromanometer catheter in the proximal outflow graft (A) with corresponding CT locations (B). Hemodynamic waveforms showed dynamic obstruction with sitting at 3000 RPM with a reduced outflow gradient (C), whereas without dynamic inflow obstruction at 2600 RPM, there was only residual fixed obstruction in the outflow cannula and no evidence of a flow-mediated augmentation in gradient due to unkinking of the outflow graft (D). RPM - revolutions per minute.

Simultaneously, to assess the outflow graft, a multipurpose guide catheter was threaded into the aorta, while a high-fidelity micromanometer wire was advanced into the outflow graft beyond the kink, shown fluoroscopically in Figure 3A (analogous locations on CT are indicated in Figure 3B). At 3000 RPM, a pressure drop across the obstruction was measured at 20 mmHg, which when repeated in a sitting position was 16 mmHg (Figure 3C). There was a dramatic fall in both pressure waveforms due to a decrease in the flow. However, the drop in peak-to-peak pressure gradient across the outflow cannula or drop in absolute pressures were not demonstrated at 2600 RPM (Figure 3D), suggesting that there was no flow deficit with sitting at this speed and that there was no significant unkinking, which could manifest as an increased pressure gradient due to higher flow. Therefore, changes in the outflow graft obstruction were unlikely to increase the risk of suction events, and no intervention was undertaken. Ultimately, orthostatic symptoms improved with speed changes, and hemodynamics were managed with medical therapy, but intermittent symptomatic ventricular arrhythmias continued requiring ventricular tachycardia ablation.

## Discussion

The present case demonstrates the imperative to fully evaluate hemodynamics in symptomatic patients supported on LVAD therapy. Dynamic inflow cannula obstruction can be difficult to appreciate, particularly if position-dependent and in patients with challenging echocardiographic images. The most important clues to this etiology of paroxysmal symptoms were the prominent decrease in left ventricular dimensions with position change and on echocardiography a decrease in inflow cannula velocities [6,7].

With dynamic symptomatology, inflow obstruction, and 3D reconstruction of the outflow graft concerning hemodynamically significant obstruction with poor baseline hemodynamics, further evaluation was required to delineate the significance of each pathology. The present case conjured a unique pathophysiologic mechanism for suction: that unkinking of the outflow graft may have resulted in significantly improved flow due to the afterload sensitivity of the pump, contributing to suction events. This diagnostic challenge prompted an invasive investigation, which confirmed that dynamic inflow obstruction was solely responsible for the clinical findings.

Outflow kinking is a difficult complication to manage effectively, as even if identified invasively, there is a lack of data about criteria for intervention based on outflow graft hemodynamics [1]. Further studies are needed to understand the hemodynamic significance of outflow cannula graft obstructions.

## Conclusions

The importance of thorough hemodynamic evaluation is critical for evaluating symptoms, particularly when dynamic and multiple possible etiologies exist. Given the known limitations of surface echocardiography, consideration should be given to intracardiac echocardiography and invasive hemodynamic assessment may be warranted.
